# Supplementary material for: Combination Therapy with a TLR7 Agonist and a BRD4 Inhibitor Suppresses Tumor Growth via Enhanced Immunomodulation
Source: Int J Mol Sci. 2024 Jan 4;25(1):663. doi: 10.3390/ijms25010663 (PMC10779224; doi:10.3390/ijms25010663)
Supplement: Supplementary file 1 [file ijms-25-00663-s001.zip › ijms-2768191-supplementary.pdf]

Supplementary Material

1 Supplementary Figures

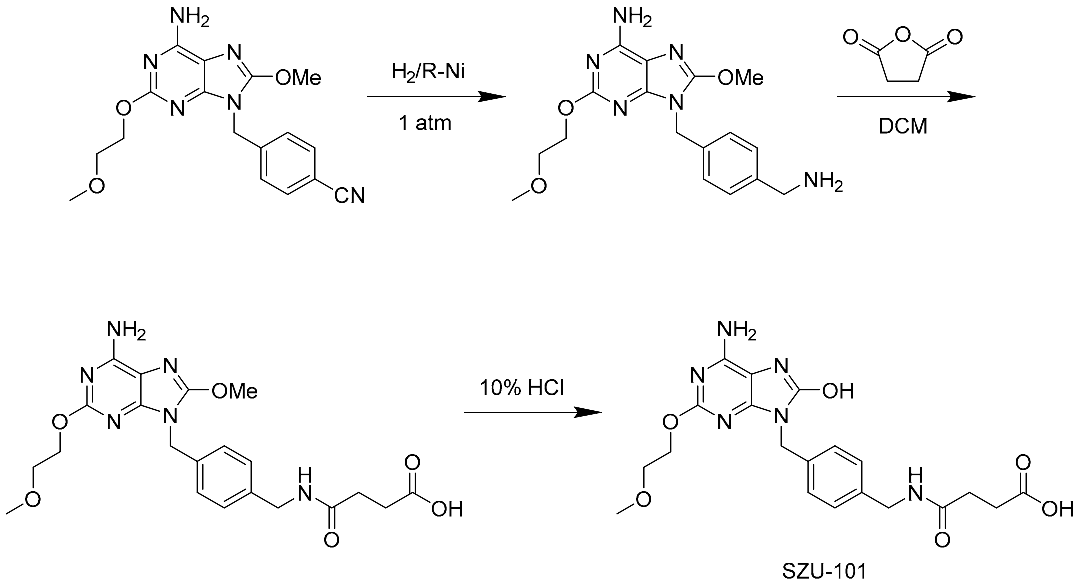

Supplementary Figure S1. Schematic illustration of the synthesis of SZU-101.

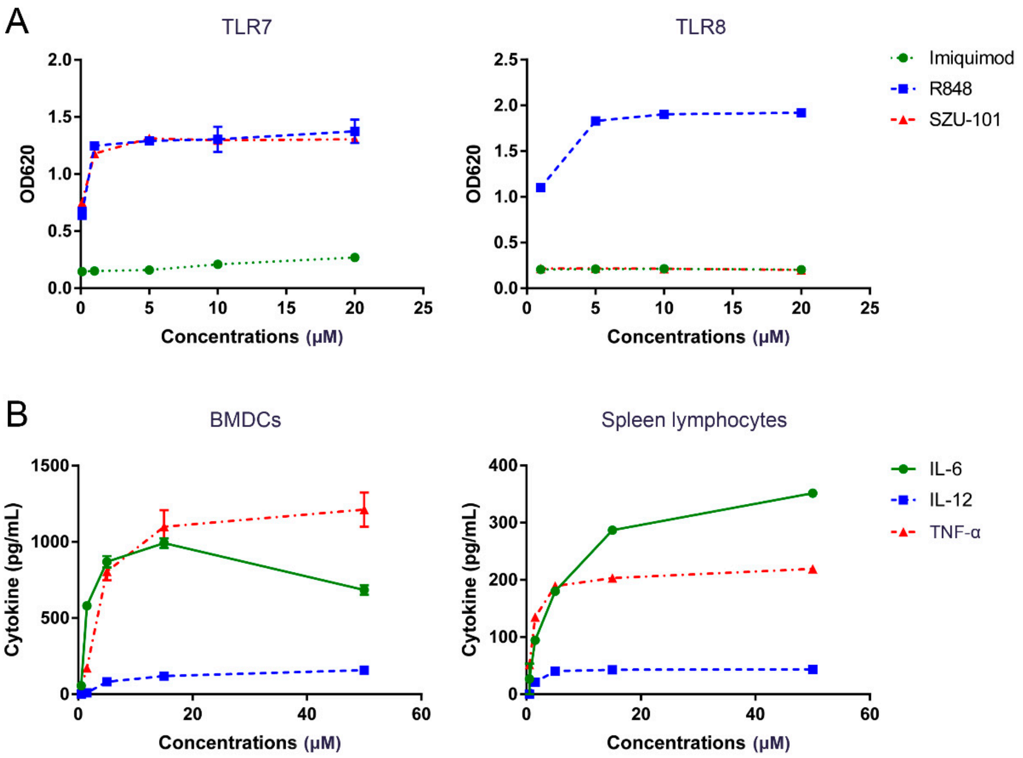

Supplementary Figure S2. SZU-101 stimulated TLR7 signaling and primed immune responses in vitro. (A) HEK-Blue hTLR7 and hTLR8 cells were treated with SZU-101 for 24 h, and final OD values were recorded at 620 nm. (B) Mouse BMDCs and spleen lymphocytes were treated with SZU-101 for 24 h, and cytokine levels (IL-6, IL-12 and TNF-α) were quantified by ELISA. Data represent mean ± SE (n = 3).

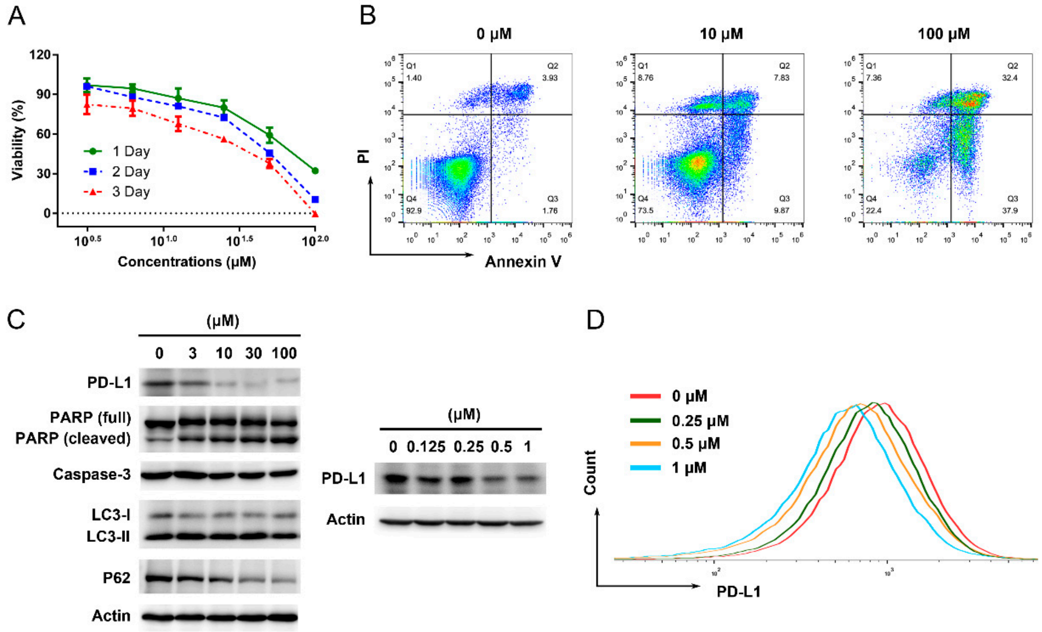

Supplementary Figure S3. JQ-1 displayed growth inhibition on 4T1 cells in vitro. (A) Growth inhibition on 4T1 cells treated by JQ-1 for 1-3 days, determined by CCK-8 assay. (B) Annexin V-FITC/PI analysis of 4T1 cells treated by JQ-1 for 24 h. (C) Detection of the proteins of 4T1 cells treated by JQ-1 for 24 h, determined by western blot, including PD-L1, PARP, Caspase-3, LC3B and P62. (D) Detection of PD-L1 of 4T1 cells treated by JQ-1 for 24 h, determined by flow cytometry. Data represent mean ± SE (n = 3).

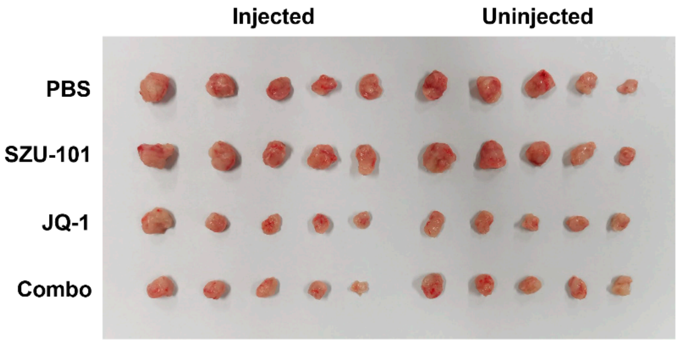

Supplementary Figure S4. Representative images of the excised tumors of 4T1-bearing Balb/c mice treated with SZU-101 and JQ-1.

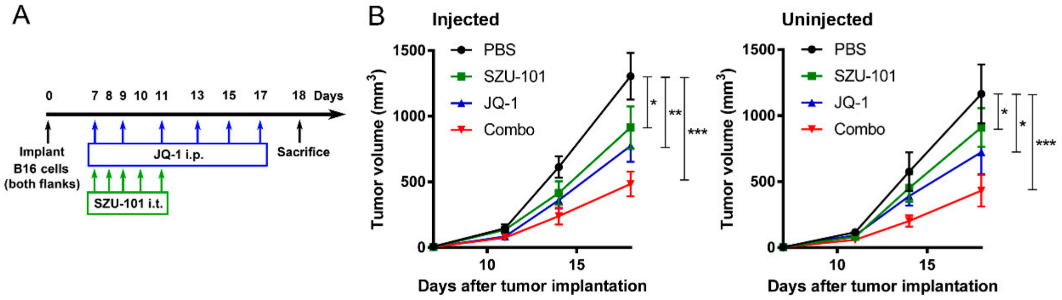

Supplementary Figure S5. Combination administration of SZU-101 and JQ-1 inhibited melanoma growth at both injected and uninjected sites. (A) Experimental protocol of combination therapy with SZU-101 and JQ-1. C57BL/6J mice (*n* = 5-6/group) were implanted with $2{\times}10^5$ B16 cells in both flanks, and i.t. treated with SZU-101 and i.p. treated with JQ-1. (B) Tumor volumes at both injected and uninjected sites were monitored. Data represent mean ± SE, * $P<0.05$, ** $P<0.01$, *** $P<0.001$ (two-way ANOVA with Bonferroni post hoc test).

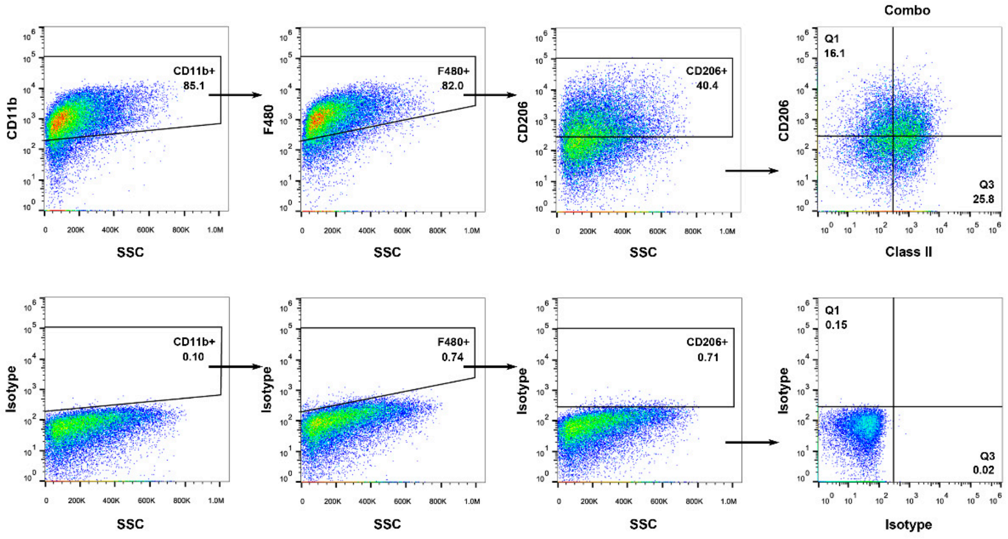

Supplementary Figure S6. Representative flow cytometric plots of M1 and M2 macrophages in TAMs. The single cell suspensions of tumors were prepared and stained for CD45, CD11b and F4/80 to identify TAMs. Tumor infiltrating M1 and M2 macrophages were identified as $CD206^-$ and $CD206^+$ populations in TAMs, respectively. M1 and M2 macrophages were further identified as $CD206^-$Class II^+ and $CD206^+$Class II^- populations, respectively. Representative plots of cells after staining with isotype antibodies were shown on the lower panel.

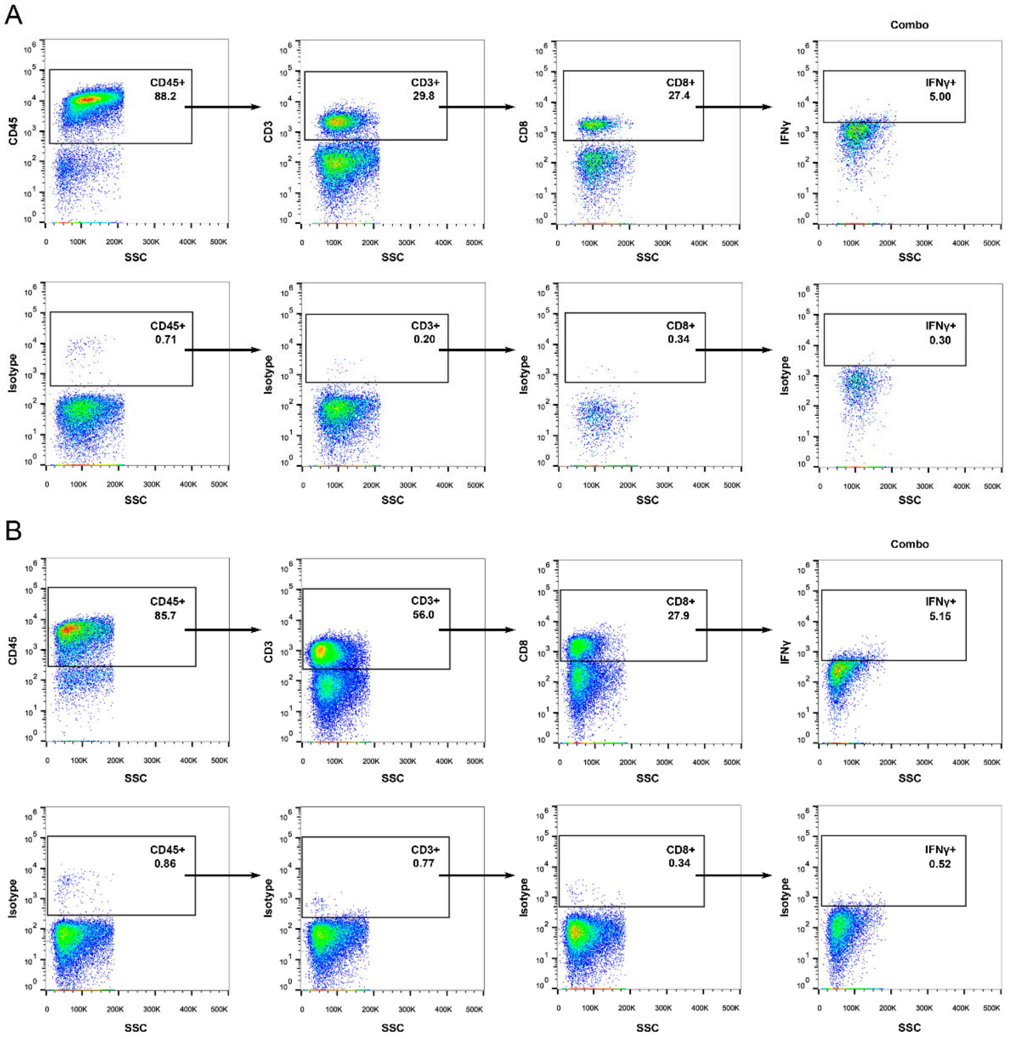

Supplementary Figure S7. Representative flow cytometric plots of $CD8^+$ T cells in spleens (A) and TILs (B). The single cell suspensions of spleens and tumors were prepared and stained for CD45, CD3, CD8 and intracellular IFNγ to identify $CD8^+IFN\gamma^+$ T cells. Representative plots of cells after staining with isotype antibodies were shown on the lower panel.

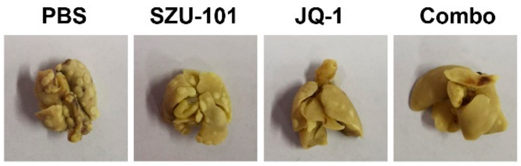

Supplementary Figure S8. Representative images of the excised lungs of 4T1-bearing Balb/c mice treated with SZU-101 and JQ-1.

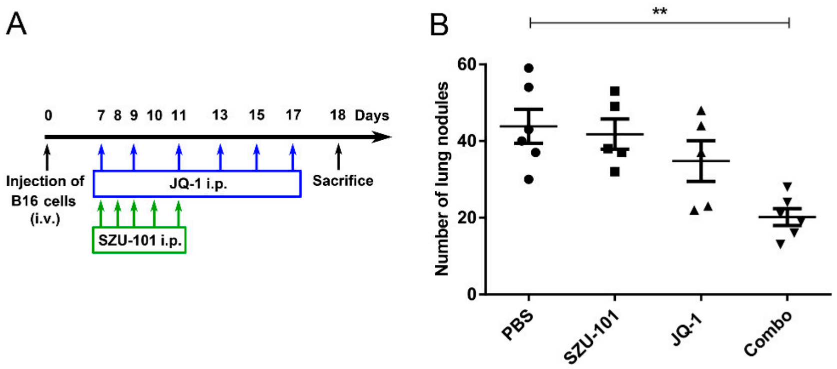

Supplementary Figure S9. Combination administration of SZU-101 and JQ-1 inhibited melanoma metastasis. (A) Experimental protocol of tumor lung metastasis. C57BL/6J mice (*n* = 5-6/group) were intravenously injected through the tail vein with 5×10^4 B16 cells on Day 0, and i.p. treated with SZU-101 and JQ-1. (B) Numbers of lung nodules were counted on Day 18. Data represent mean ± SE, ** *P*<0.01 (one-way ANOVA with Tukey's post hoc test).

2 Supplementary Table

Supplementary Table S1. Supplementary Table I Antibodies used in flow cytometry analysis.

Antibody	Color	Cat#	Source
CD45	BV605	103140	BioLegend
CD3	FITC	100204	BioLegend
CD8	PerCP/Cy5.5	100734	BioLegend
PD-L1	APC	124312	BioLegend
IFN-γ	BV421	505830	BioLegend
CD11b	FITC	101206	BioLegend
F4/80	PerCP/Cy5.5	123128	BioLegend
CD206	APC	141708	BioLegend
MHC Class II	APC/Cy7	107628	BioLegend
